# Differences in Cognitive-Perceptual Factors Arising From Variations in Self-Professed Paranormal Ability

**DOI:** 10.3389/fpsyg.2021.681520

**Published:** 2021-06-10

**Authors:** Kenneth Graham Drinkwater, Neil Dagnall, Andrew Denovan, Christopher Williams

**Affiliations:** Department of Psychology, Manchester Metropolitan University, Manchester, United Kingdom

**Keywords:** delusion formation, thinking style, paranormal ability and belief, reality testing, emotion-based reasoning, belief in science

## Abstract

This study examined whether scores on indices related to subclinical delusion formation and thinking style varied as a function of level of self-professed paranormal ability. To assess this, the researchers compared three groups differing in personal ascription of paranormal powers: no ability, self-professed ability, and paranormal practitioners (i.e., Mediums, Psychics, Spiritualists, and Fortune-Tellers). Paranormal practitioners (compared with no and self-professed ability conditions) were expected to score higher on paranormal belief, proneness to reality testing deficits, emotion-based reasoning, and lower on belief in science. Comparable differences were predicted between the self-professed and no ability conditions. A sample of 917 respondents (329 males, 588 females) completed self-report measures online. Multivariate analysis of variance (MANOVA) revealed an overall main effect. Further investigation, using discriminant descriptive analysis, indicated that paranormal practitioners scored higher on proneness to reality testing deficits, paranormal belief, and emotion-based reasoning. Belief in science did not meaningfully contribute to the discriminant function. Overall, results were consistent with previous academic work in the domains of paranormal belief and experience, which has reported that paranormal-related cognitions and perceptions are associated with factors related to subclinical delusion formation (i.e., emotion-based/intuitive thinking).

## Introduction

Surveys report that belief in the paranormal endures within modern Western societies (Ipsos MORI, 1998, 2003; Newport and Strausberg, 2001; Moore, 2005). They note also that people frequently disclose paranormal experiences (Dagnall et al., 2016). This observation aligns closely with the classic work of significant parapsychological researchers such as Wilhelm Heinrich Carl Tenhaeff and Sybo Schouten (e.g., Tenhaeff, 1972; Schouten, 1994), who observed that paranormal experiences were an important feature of existence meriting empirical investigation. Despite interest in belief and experience, relatively few academic studies have examined psychological factors associated with self-professed paranormal ability. Those that have, note that ascription of ability is linked to certain cognitive-perceptual characteristics. For instance, Krippner et al. (1998) examined the psychological profiles of several participants who had undertaken consciousness training delivered by Ramtha, a discarnate entity. Psychological tests indicated that participant profiles were characterised by thin boundaries and high levels of absorption (i.e., susceptibility to immersive and self-altering experiences) and dissociation (i.e., temporary inhibition of threatening perceptions/memories). Typically, individuals with thin boundaries do not repress or isolate uncomfortable material. These elements manifest as creativity and the tendency to get lost in fantasy. Relatedly, Parra and Carlos Argibay (2012) found that alleged psychics (vs. controls) scored higher on dissociation, absorption, and fantasy proneness.

Under reporting of self-professed supernatural powers is counter-intuitive because prominent paranormal measurement instruments have historically acknowledged the importance of ability alongside belief and experience. For instance, the Australian Sheep Goat Scale (Thalbourne and Delin, 1993), which is one of the most used instruments to assess belief in the paranormal, contains items indexing belief (e.g., “I believe in the existence of ESP”), experience (e.g., “I believe I have had personal experience of ESP”), and ability (e.g., I believe I am psychic). Similarly, the Anomalous Experiences Inventory (AEI) comprises subscales assessing Anomalous/Paranormal Experiences, Beliefs, Fear, and Ability (Gallagher et al., 1994).

The inclusion of self-professed ability demonstrates that it is often conceptualised as a significant facet of paranormality (i.e., the state of being paranormal). Although, it is important to note that while ability is related to belief and experience, it also represents a discrete construct. This is illustrated by consideration of zero-order correlations between AEI subscales, which reveal a series of medium-large associations. Indeed, Ability shares only 15% variance with Belief and 38% with Experiences. Ability also relates differently to variables such as neuroticism and general sensation seeking. In both cases, Experiences and Beliefs correlate positively, whereas there is no relationship with Ability (Gallagher et al., 1994).

Despite widescale belief in the paranormal and relatively frequent reporting of concomitant experiences, there remains only limited, highly contested empirical evidence to support the existence of supernatural phenomena (Bressan, 2002; Dagnall et al., 2007). Acknowledging this, from a rational, scientific standpoint, critics, and sceptics contend that the designation of paranormal ability arises from mundane conventional processes. From a psychological perspective, a prevailing explanation for self-professed psychic ability is faulty attribution. Specifically, the misattribution of event outcome (s) to mysterious forces, powers, and entities (Wiseman and Watt, 2006).

This explanation is consistent with Irwin et al. (2013), who posit that personal validation of paranormality arises from two distinct but connected stages. Stage one involves conscious awareness of an inexplicable anomaly (unusual occurrence), in the form of a stimulus or event for which there is no apparent, rational explanation. Stage two requires the percipient to assign paranormal causation to the occurrence (Lange et al., 2019). Thus, the reification of paranormal ability results from personal interpretation, and the desire to comprehend anomalous events and incidences. Once established, the attributional framework structures ensuing happenings and stimuli. This aligns with the notion of worldview, which is a set of high order beliefs that influence appraisal of existence and reality (Zusne and Jones, 1982, 1989; Koltko-Rivera, 2004; Dagnall et al., 2015). Accordingly, belief in paranormal ability facilitates the search for outcomes and evidence that validate the supposition of “ability” and negate inconsistent information. Consequently, paranormal believers tend to demonstrate susceptibility to confirmation bias (Drinkwater et al., 2012; Irwin et al., 2012b).

The notion that self-professed psychic ability in the general population arises from active misinterpretation of causation, is consistent with the notion that uncorroborated paranormal inferences represent sub-clinical delusions (e.g., van Os, 2003; Unterrassner et al., 2017). Hence self-professed psychic ability in the absence of empirical support, is located on the same continuum as full-blown psychosis, and accordingly manifests similar features such as odd beliefs/behaviours and anomalous perceptions (Unterrassner et al., 2017).

Furthermore, a defining feature of delusions is that they are formed without due consideration of alternatives and lack rigorous, rational scrutiny of the evidence from which they derive (Diagnostic and Statistical Manual of Mental Disorders, Fifth Edition, DSM-5; American Psychiatric Association, 2013). Corresponding with this definition a key characteristic of delusional thinking is inadequate reality testing. Thus, delusions are rigid beliefs that persist in the face of contradictory evidence (Irwin et al., 2012a). Noting this, researchers have explained belief in the paranormal in terms of reality testing failures (Irwin, 2003, 2004). These are best defined as deficiencies in the capacity to differentiate self from non-self, intrapsychic from external stimuli, and to maintain empathy with ordinary social criteria of reality (Kernberg, 1996).

Irwin et al. (2012b) contend that another important feature of delusions is that they are persistent beliefs endorsed on their emotional (rather than) rational appeal. This concurs with the clinically informed notion of delusions as beliefs arising from faulty interpretation of anomalous experiences (Garety and Freeman, 1999), and/or inadequate evidence (Coltheart et al., 2010; Irwin et al., 2012b). The relationship between emotion-based reasoning and belief in the paranormal is well-established (Sappington, 1990; Irwin et al., 2012a).

Proneness to reality testing deficits and inclination to emotion-based reasoning are also principal characteristics of intuitive-experiential thinking (Denovan et al., 2017). This thinking style features in various dual processing models. Although these differ, they agree on the concept that thinking/information processing is best conceptualised as two fundamentally different modes. A prominent dual processing model that researchers have applied to the study of paranormal belief and experience is cognitive-experiential self-theory (CEST; Epstein et al., 1996). Congruent with subclinical delusions the experiential system is self-evidently valid; based on associationistic connections; automatic, affective oriented; mediated by experience; resistant to change; holistic; encodes reality in concrete images, metaphors, and narratives. In contrast, the rational system is driven by external, objective evidence; based on logical connections; intentional; reason oriented; mediated by conscious appraisal of events; analytical; and encodes reality in abstract symbols, words, and numbers.

Accordingly, these two systems are associated with different preferential processing styles. Individuals high in experiential thinking place an emphasis on intuitive (subjective based) data, whereas greater levels of rational thinking manifest as a drive for analytical (objective based) evidence. Consistent with the dual processing framework, studies have robustly demonstrated that intuitive processing is positively correlated with paranormal belief (e.g., Irwin and Young, 2002; Aarnio and Lindeman, 2005; Dagnall et al., 2017) and experience (Drinkwater et al., 2020). Although some researchers report that increased critical thinking reduces belief in the paranormal (Barberia et al., 2018; Wilson, 2018), others have found no effects (Irwin et al., 2013). Noting this, instead of a measure of critical thinking, the present paper included the Belief in Science Scale (Farias et al., 2013) as an index of predilection for objective (vs. subjective) evidence.

## The Present Study

Drawing on preceding research, this study investigated whether scores on indices related to subclinical delusion formation and thinking style varied as a function of level of self-professed paranormal ability. To ensure that the full range of ability was considered, paranormal practitioners (i.e., Mediums, Psychics, Spiritualists, and Fortune-Tellers) were compared with those declaring ability, and respondents with no stated supernatural facilities. The focus on perceived ability and graded comparisons between levels of self-professed ability (practitioners vs. personally endorsed supernatural powers) signified a novel and innovative approach. It was hypothesised that paranormal practitioners would score higher on variables associated with delusional and intuitive thinking (i.e., paranormal belief, proneness to reality testing, and emotion-based reasoning) and lower on an index of analytical-rational (i.e., belief in science) than the ability group, who in turn should differ similarly to the no ability condition.

Consideration of paranormal practitioners was important because they are typically sincere in their beliefs. Particularly, they represent a group of people who not only believe they possess paranormal abilities, but also consider themselves able to produce their psychic powers in a systematic, focused manner. This active approach generally contrasts with people professing ability, as they generally view their abilities as less defined and more spontaneous. These factors suggest that psychic practitioners will demonstrate higher levels of confidence in their abilities than those who merely profess supernatural powers.

## Method

### Respondents

The sample consisted of 917 respondents (Mean age, *M*) = 33.25 years, *SD* = 14.75, range 18–83. There were 329 males (36%), *M* = 37.34 years, *SD* = 15.55, range 18–82; and 588 females (64%), *M* = 30.97 years, *SD* = 13.78, range 18–83. Respondent recruitment occurred through Bilendi, an online data management platform. The only exclusion criterion was that respondents must be at least 18 years of age.

### Measures

#### Self-Professed Paranormal Abilities (Drinkwater et al., 2020)

Respondents completed a series of items determining whether they were paranormal practitioners (i.e., Medium, Psychic, Spiritualist, and Fortune-Teller). Each category contained a definition (i.e., Mediums, possess the ability to receive, and relay information from deceased people to the living; Psychics, perceive energy left behind from people who have died; Spiritualists, provide information regarding the transition of the human spirit from the physical body to the afterlife; or Fortune-Tellers, have the ability to foretell future event). This was followed by a “Yes/No” option, and an item asking respondents to indicate the extent to which they believed they possessed each ability (0–100 percent). In addition, respondents could also indicate “Other” if their self-professed ability fell outside the cited categories. Collectively, these items embody core receptive elements of belief in the paranormal (Drinkwater et al., 2018).

#### Belief in the Paranormal

Belief in the paranormal was assessed using the Manchester Metropolitan University New (MMU-N). Several previous studies have used this scale (Dagnall et al., 2014; Drinkwater et al., 2020). The MMU-N contains 50-items related to (haunting, superstition, religious belief, alien visitation, extrasensory perception, psychokinesis, astrology, and witchcraft) (Dagnall et al., 2010a,b; Dagnall et al., 2011). This measure was employed in preference to the widely used Revised Paranormal Belief Scale (Tobacyk, 2004) and Australian Sheep Goat Scale (Thalbourne and Delin, 1993) because it samples greater intra dimension phenomena and a wider range of construct content (Dagnall et al., 2010a,b). Respondents responded via a seven-point Likert scale (ranging from 1, strongly disagree, to 7, strongly agree). The MMU-N has demonstrated excellent internal reliability and validity (face and concurrent), indicating that it is both conceptually coherent and psychometrically robust (Drinkwater, 2017). In this study, the MMU-N demonstrated excellent internal reliability, α = 0.96.

#### Reality Testing

The reality testing subscale of the Inventory of Personality Organization (IPO-RT; Lenzenweger et al., 2001) measures the capacity to differentiate self from non-self, intrapsychic from external stimuli, and maintain empathy with ordinary social criteria of reality (Kernberg, 1996). Commensurate with this delineation, the IPO-RT is an established scale for assessing preference for intuitive-experiential processing (subjective thinking) (Denovan et al., 2017), and proneness to reality testing deficits (Irwin, 2004; Drinkwater et al., 2012; Dagnall et al., 2017). The IPO-RT comprises 20-items presented as statements (e.g., “When I'm nervous or confused, it seems like the things in the outside world don't make sense either”). Respondents indicated agreement via a five-point Likert scale (1 = never true to 5 = always true). Higher scores are indicative greater reliance on intrapsychic activity (i.e., intuitive-experiential thinking) (Dagnall et al., 2018). The IPO-RT possesses good internal and external reliability, and construct validity (Lenzenweger et al., 2001; Drinkwater et al., 2012; Dagnall et al., 2018). IPO-RT possessed excellent internal reliability in the current study, α = 0.93.

#### Belief in Science

The Belief in Science Scale (BISS; Farias et al., 2013) evaluates the degree to which science is valued as a source of superior knowledge. The scale comprises ten statements (e.g., “The only real kind of knowledge we can have is scientific knowledge”). Respondents specify agreement using a six-point Likert scale (1 = Strongly Disagree to 6 = Strongly Agree). Thus, total scale scores range from 10 to 60; these are expressed as an average (1.0–6.0). Higher scores indicate greater faith in the scientific approach. The BISS performs well-psychometrically; possesses validity, and high internal consistency (Farias et al., 2013; Dagnall et al., 2019). High alpha reliability was evident in this study, α = 0.92.

#### Emotion-Based Reasoning

The degree to which decision-making is based upon affective reactions was measured using the Emotion-Based Reasoning (EBR) subscale of the Cognitive Biases Questionnaire (CBQ) (Peters et al., 2014). The EBR comprises 6-items, framed within brief vignettes. Respondents specify one of three options that best epitomises their feelings. Options are recorded on a three-point scale and include, 1 = absence of bias; 2 = presence of bias with some qualification; and 3 = presence of bias. Totalling EBR items produced a score ranging from 6 to 18; high scores represent greater affect-based decision-making. The CBQ has established psychometric properties (e.g., internal reliability, *r* = 0.89; and test-retest reliability, *r* = 0.92) (Peters et al., 2014). In the present study, the EBR produced an acceptable alpha coefficient of α = 0.61 (Nunnally and Bernstein, 1994; Ursachi et al., 2015).

### Procedure

Potential respondents clicked a web-link to receive study information. After providing informed consent, respondents then progressed to the actual online survey. At this point, instructions informed respondents to carefully read and complete all questions, respond honestly, and work through items at their own pace. The survey comprised sections demographic characteristics (i.e., age and preferred gender), self-professed paranormal abilities, and the measurement scales (i.e., MMU-N, BISS, IPO-RT, and EBR). Sections and measurement scales rotated across respondents to prevent order effects. At the end of the survey respondents received the debrief.

Data collection occurred at one point in time. A frequent criticism of this cross-sectional approach is its proneness to common method variance (CMV) (Spector, 2019). To prevent CMV the study employed procedural remedies (Krishnaveni and Deepa, 2013). Explicitly, section instructions created psychological distance between scales by accentuating differences between constructs and measures (Podsakoff et al., 2003). Moreover, instructions reduced the potential for evaluation apprehension and social desirability effects by telling respondents that there were no right or wrong responses, and that they should answer questions honestly.

### Ethics Statement

The Manchester Metropolitan University Faculty of Health, Psychology and Social Care Ethics Committee (October 2018) provided ethical approval for a series of studies examining psychological and neuropsychological factors associated with self-professed psychic ability/mediumship.

## Results

### Preliminary Analyses

Initial data examination used the professed ability (i.e., ability vs. no ability) and practitioner group (i.e., Mediumship, Psychics, Spiritualists, and Fortune-Telling) responses. This provided comparisons in terms of frequencies and percentages (see Table 1), and revealed that the number of paranormal practitioners was consistent across specialities (i.e., Mediumship, *n* = 17; Psychic, *n* = 19; Spiritualist, *n* = 22; and Fortune-Tellers, *n* = 14).

**Table 1:** Frequencies (and percentages in brackets) of professed ability and practitioner groups.

						**Ability Ratings**			
	**Ability**		**Status**			**Practising**		**Non-practising**	
**Practitioner Group**	**Yes**	**No**	**Practising**	**Non-practising**	**Total**	***M***	***SD***	***M***	***SD***
Mediumship	285 (31.1)	632 (68.9)	17 (6.0)	268 (94.0)	285	60.59	33.81	35.52	26.21
Psychic	299 (32.6)	618 (64.7)	19 (6.4)	280 (93.6)	299	61.58	28.73	35.96	25.91
Spiritualist	255 (27.8)	662 (72.2)	22 (8.6)	233 (91.4)	255	59.55	34.15	35.92	25.73
Fortune-Teller	244 (26.6)	673 (73.4)	14 (5.7)	230 (94.3)	244	55.71	28.48	31.38	23.71

Practitioners (vs. non-practising) demonstrated greater confidence in their self-professed paranormal abilities. It was not, however, possible to test this difference because practitioners differed in the number of services they provided (practised in one domain, *n* = 16, 46%; two domains, *n* = 6, 17%; three domains, *n* = 8, 23%; and all four domains, *n* = 5, 14%). Moreover, it was difficult to interpret this trend since it was unclear whether practising resulted in higher confidence, or greater conviction informed the motivation to practise.

Subsequently, to produce an overall condition for comparison with non-practising and no ability conditions, it was necessary to combine the practitioner groups. Collapsing practitioner groups revealed, *n* = 35(4% of the total sample) were practising, *n* = 373 (41%) non-practising, and *n* = 509 (56%) no self-professed ability.

Descriptive information for paranormal belief, reality testing, emotion-based reasoning, and belief in science scores as a function of ability condition appears in Table 2. The practising group scored highest on belief in the paranormal, proneness to reality testing deficits and emotion-based reasoning, followed by the non-practising and the no ability groups. The opposite trend existed for belief in science.

**Table 2:** Means and standard deviations for paranormal belief, reality testing, emotion-based reasoning, and belief in science as a function of ability.

		**Variable**							
		**Paranormal belief**		**Belief in science**		**Reality testing**		**Emotion-based reasoning**	
**Ability**	***N***	***M***	***SD***	***M***	***SD***	***M***	***SD***	***M***	***SD***
Practising	35	226.14	43.02	36.05	11.32	61.34	18.50	10.0	2.12
Ability	373	200.65	43.38	36.88	10.12	48.64	13.52	9.09	2.25
No ability	509	151.63	52.08	40.80	11.84	36.26	10.91	7.53	1.73

### Comparisons

Multivariate analysis of variance (MANOVA) tested whether ability scores (no ability, ability, practicing) were significantly different across measures. Prior to performing MANOVA, data screening occurred. Consideration of skewness and kurtosis indicated satisfactory results (i.e., all values fell within the range of −2 to +2; Byrne, 2010). Data points signified outliers if they had an absolute studentized residual >4 and a Cook's distance > 4 / (*n*–*k*−1), where *n* is the sample size and *k* is the quantity of independent variables. No outliers existed using these criteria. However, examining homogeneity of variance-covariance matrices *via* Box's test revealed a significant result (144.34, *p* < 0.001), possibly due to unequal cell sizes. When this occurs, Tabachnick et al. (2007) recommend using the more robust Pillai's criterion rather than Wilk's lambda.

The results of the MANOVA revealed a significant main effect of ability scores on paranormal belief, reality testing, emotion-based reasoning, and belief in science, Pillai's criterion = 0.33, *F*_(8, 1824)_ = 44.99, *p* < 0.001. A medium effect size existed, η^2^ = 0.17. A *post-hoc* descriptive discriminant analysis examined how ability conditions manifested across paranormal belief, reality testing, emotion-based reasoning, and belief in science. One significant discriminant function existed, λ = 0.67, $\chi_{\left(8\right)}^{2}$ = 361.69, *p* < 0.001. This explained 98.4% of variance, canonical *R*^2^ = 0.57. According to the standardized canonical discriminant functions and structure coefficients (Table 3), reality testing, paranormal belief and emotion-based reasoning were important in discriminating between levels of ability. Specifically, all evidenced structure coefficients >0.32 (Tabachnick et al., 2007). Reality testing contributed most to group separation (i.e., the discriminant function), followed by paranormal belief, and emotion-based reasoning. Centroid means indicated that the practicing condition (1.59) exhibited greater levels of reality testing, paranormal belief, and emotion-based reasoning than the ability (0.66) and no ability (−0.59) conditions.

**Table 3:** Standardized canonical discriminant function coefficients and structure matrix of ability.

**Variable**	**Co-efficient**	***r_***s***_***
Reality testing	0.54	0.83
Paranormal belief	0.50	0.78
Emotion-based reasoning	0.25	0.61
Belief in science	−0.05	−0.26

## Discussion

Analysis revealed differences between self-professed ability groups on belief in the paranormal, proneness to reality testing deficits, and emotion-based reasoning. Specifically, paranormal practitioners possessed higher scores on these variables compared with self-professed ability and no ability groups. The no ability group evidenced the lowest scores. This pattern of results was consistent with the notion that paranormal practitioners score higher on indirect indices of delusion proneness and propensity to intuitive thinking (Irwin et al., 2012a,b). These outcomes also generally aligned with study hypotheses. Belief in science, however, did not meaningfully contribute to differences in self-professed ability.

Overall, these results concurred with studies that have identified a typical profile associated with paranormal-related cognitions and perceptions (see Krippner et al., 1998; Parra and Carlos Argibay, 2012). Within the current paper, this comprised higher proneness to reality testing deficits and greater reliance on emotion-based/intuitive thinking. These factors are associated with delusion formation and maintenance in subclinical populations (Irwin et al., 2012b).

The failure to observe the full range of group-based differences across comparisons for belief in science, an index of preference for objective (vs. subjective) based evidence, aligned with prior work on critical thinking (see Wiseman and Watt, 2006). Although some researchers report that increased critical thinking reduces belief in the paranormal (Barberia et al., 2018; Wilson, 2018), others have observed no effects (Irwin et al., 2013). Discrepancies may arise from the fact that studies have used an array of measures. Additionally, indirect self-report instruments, such as the Belief in Science Scale, may not accurately index formal analytical reasoning. This is because they rely on the validity of individual responses and awareness of processes that are not fully available to consciousness.

Furthermore, accuracy is difficult to establish as data is collected at a separate point in time to performance, meaning that judgments derive from memory. Additional problems are that individuals interpret questionnaire items differently; base their conclusions on readily accessed personal contexts, and information triggered within the testing situation can influence responses. These and other limitations explain why self-reported metacognitive measures often poorly predict actual performance (Jacobse and Harskamp, 2012).

Regardless, critical thinking is a difficult construct to assess due to variations in definition and conceptualisation (i.e., evidence of multidimensionality) (Bensley and Murtagh, 2012). Noting these concerns, alongside belief in science (i.e., preference for objective data), future research should include direct, measures of rational processing (see Pennycook et al., 2016). This will indicate the degree to which belief in science is associated with critical thinking and determine whether these factors interact to reduce paranormal factors (belief, ability, and experience).

Factors such as these may explain why preceding academic work using the dual processing framework has found stronger and more consistent effects for intuitive-experiential thinking/processing (vs. analytical-rational). A further factor to consider, which is rarely acknowledged in prior work, is that these two systems work in parallel (Epstein et al., 1996). Thus, differences may reflect the tendency to engage in intuitive processing rather than a deficit in critical thinking. This is an important notion since it suggests that variations in thinking style are domain specific rather than general. To test this supposition, subsequent research could investigate whether self-professed paranormal ability is associated with explicit cognitive errors such as confirmation bias and misrepresentation of chance. Nonetheless, findings overall suggest that intuitive thinking plays a more important role in paranormal attributions than weaknesses in analytical-rational processing.

The present paper found that within practitioner group comparisons were conflated by the fact that individuals frequently professed multiple abilities. This indicated that future research should base contrasts on alternative distinctions. In this context, qualitative work with Mediums, Psychics, Spiritualists, and Fortune-Tellers advocates that they do not find these categorisations useful or appropriate. Instead, individuals prefer to qualify their abilities in terms of their perceived phenomenology. These decisions are frequently axiologically motivated, deriving from value judgments and ethics, and influence practice. For instance, practitioners frequently self-classify as “sensitive” or “intuitive.” Although these terms lack precise operationalisation, they denote different paranormal attributions. Sensitives are receptive to psychic information, which they interpret, whereas intuitives believe that they possess awareness of things that have or could occur. Practitioners also make distinctions between spontaneous and controlled abilities (external vs. internal). Subsequent studies could better define these terms and examine whether these perceived distinctions manifest as profile variations. This seems possible, as practitioners perceive paranormal phenomena in diverse ways. Key examples being a strong reliance on either visual or auditory stimuli.

This approach will enable the identification of important dissimilarities in the psychological and psychopathological profiles of paranormal practitioners. This will potentially reveal whether high self-professed believers represent a homogeneous group. Another research advance is to combine self-professed ability with factors likely to reinforce convictions. For example, confidence ratings of own psychic facilities, length and breadth of practice, perceived success, client numbers, etc. Identifying factors that predict self-professed ability would enable the development of class profiles. These, combining key attributes, would allow researchers to determine whether practitioners differ in subtle, yet important ways.

A potential criticism of the present study was that it focussed on only a restrictive range of paranormal practises. These were selected because they reflected psychic phenomena associated with anomalous mental phenomena (psi) and communication with the dead (psychic occurrence, mediumship, spiritualism, telepathy, precognition, premonition, etc.), and represented core tenets of parapsychology as defined by the Society for Psychical Research (SPR). The SPR focuses on the nature and causes of psi and survival research (i.e., whether aspects of consciousness or personality survive bodily death). These elements are correspondingly encapsulated within the Australian Sheep Goat Scale, which is a frequently used measure of paranormal belief (Drinkwater et al., 2018). In this context, the practice areas were well-grounded, and sampled adequate domain content.

Acknowledging the existence of practitioners in other paranormal and scientifically unsubstantiated areas (i.e., pseudoscience and New Age Philosophy), follow-up studies should investigate whether these individuals possess similar psychological characteristics to those reported in this article. Consideration of these areas is important because they derive from different beliefs that may influence propensity to subclinical delusion formation and thinking style. For example, endorsement of pseudoscience derives from the incorrect assumption that a phenomenon is supported by the scientific method (Hines, 1988). Contrastingly, New Age Philosophy originates from the notion that humans are entering into a time of higher-level consciousness that will transform individuals and society, and rejects science, realism, and objectivity (Sjöberg and Wåhlberg, 2002). This line of work could also examine if training increases faith in ability and this expresses as heightened delusion proneness.

## Data Availability Statement

The raw data supporting the conclusions of this article will be made available by the authors, without undue reservation.

## Ethics Statement

The studies involving human participants were reviewed and approved by the Manchester Metropolitan University Faculty of Health, Psychology and Social Care Ethics Committee. The patients/participants provided their written informed consent to participate in this study.

## Author Contributions

KD and ND provided the theoretical focus, developed content, and produced the initial article. KD was responsible for data collection. AD performed data analysis and wrote up the results. All authors were involved in the final submission of the article.

## Conflict of Interest

The authors declare that the research was conducted in the absence of any commercial or financial relationships that could be construed as a potential conflict of interest.
